# Identification of guanine nucleotide-binding protein γ-7 as an epigenetically silenced gene in head and neck cancer by gene expression profiling

**DOI:** 10.3892/ijo.2013.1808

**Published:** 2013-02-06

**Authors:** SEMRA DEMOKAN, ALICE Y. CHUANG, XIAOFEI CHANG, TANBIR KHAN, IAN M. SMITH, KAVITA M. PATTANI, SANTANU DASGUPTA, SHAHNAZ BEGUM, ZUBAIR KHAN, NANETTE J. LIEGEOIS, WILLIAM H. WESTRA, DAVID SIDRANSKY, WAYNE KOCH, JOSEPH A. CALIFANO

**Affiliations:** 1 Departments of Otolaryngology-Head and Neck Surgery, Johns Hopkins Medical Institutions, Baltimore, MD;; 2 Dermatology, Johns Hopkins Medical Institutions, Baltimore, MD;; 3 Pathology, Johns Hopkins Medical Institutions, Baltimore, MD;; 4 Milton J. Dance Head and Neck Center, Greater Baltimore Medical Center, Baltimore, MD, USA;; 5 Department of Basic Oncology, Oncology Institute, Istanbul University, Capa, Istanbul, Turkey;; 6 Department of Human and Molecular Genetics, Virginia Commonwealth University, Richmond, VA, USA

**Keywords:** guanine nucleotide-binding protein γ-7, gene expression, silencing, head and neck squamous cell carcinoma, epigenetics

## Abstract

Silencing of tumor suppressor genes plays a vital role in head and neck carcinogenesis. Aberrant hypermethylation in the promoter region of some known or putative tumor suppressor genes occurs frequently during the development of various types of cancer including head and neck squamous cell carcinoma (HNSCC). In this study we used an expanded mRNA expression profiling approach followed by microarray expression analysis to identify epigenetically inactivated genes in HNSCC. Two HNSCC cell lines were treated with 5-aza-2′-deoxycytidine followed by microarray analysis to identify epigenetically silenced genes in HNSCC. We found 1,960, 614 and 427 genes were upregulated in the HNSCC cell lines JHU-012, JHU-011 and the combination of both cell lines, respectively. HNSCC tumor and normal mucosal samples were used for gene profiling by a 47K mRNA gene expression array and we found 7,140 genes were downregulated in HNSCC tumors compared to normal mucosa, as determined by microarray analysis, and were integrated with cell line data. Integrative analysis defined 126 candidate genes, of which only seven genes showed differential methylation in tumors and no methylation in normal mucosa after bisulfite sequencing. Following validation by QMSP, one gene, guanine nucleotide-binding protein γ-7 (*GNG7*), was confirmed to be highly methylated in tumors and unmethylated in normal mucosal and salivary rinse samples demonstrating cancer-specific methylation in HNSCC tissues. *TXNIP* and *TUSC2* were partially methylated in tumors and normal salivary rinses but unmethylated in normal mucosa. We concluded that *GNG7* is a highly specific promoter methylated gene associated with HNSCC. In addition, *TXNIP* and *TUSC2* are also potential biomarkers for HNSCC.

## Introduction

Among human malignancies, head and neck cancer is the sixth most common cancer in the world (1). Head and neck cancer is an aggressive and life-threatening disease with poor morbidity and high mortality in advanced disease. More than 40,000 new cases of head and neck squamous cell carcinoma (HNSCC) are diagnosed in the United States each year, with 12,000 US deaths annually. Survival rates have not improved significantly for patients with HNSCC in the past thirty years despite active clinical and basic research addressing this issue. Treatment for HNSCC includes surgical resection, chemotherapy and radiation therapy; however, approximately 50% of all patients have advanced disease at the time of diagnosis often requiring use of all three treatment modalities. Therefore, it is important to discover new biomarkers in a cancer-specific manner and to develop new methods that provide sensitive and reliable biomarkers of HNSCC for detection, treatment response and prognosis.

Genetic alterations are a hallmark of human cancer, with the activation of proto-oncogenes and inactivation of tumor suppressor genes, either through deletion or inactivating point mutations, being well defined (2). In addition to these genetic alterations, changes in DNA methylation, an epigenetic process present in mammalian cells, are also a hallmark of human cancer (3). Silencing of tumor suppressor genes by means of promoter hypermethylation plays an important role in head and neck carcinogenesis (4). Methylation of the CpG islands in the promoter regions of tumor suppressor genes is frequently observed with resultant reduced gene expression (5,6). To discover the new cancer-specific hypermethylated genes, gene expression profiling via oligonucleotide microarray-based approach is a reliable technology for whole genome epigenetic research (7). Measuring promoter hypermethylation by using real-time quantitative methylation-specific PCR (QMSP) allows an objective, robust, and rapid assessment of promoter methylation status (8–11).

Previously, we employed a pharmacologic unmasking expression array technique using a 12K gene expression array to identify epigenetically inactivated genes in HNSCC (12). In this study, we expanded this approach using a whole genome 47K array platform and then performed bisulfite DNA sequencing for 126 selected genes and QMSP for seven selected genes after evaluating the bisulfite sequencing results, to validate HNSCC-specific methylation in novel genes.

## Materials and methods

### Cell lines

We used 2 human head and neck cancer cell lines, JHU-011 and JHU-012, which were developed from a laryngeal primary and a neck node metastasis of different HNSCC patients, at the Department of Otolaryngology-Head and Neck Surgery, Johns Hopkins University. Cell lines were cultured in RPMI-1640 medium supplemented with 10% fetal bovine serum and 1% penicillin-streptomycin. All media components were obtained from Life Technologies Invitrogen Corp. All cell lines tested negative for any mycoplasma contamination.

### 5-aza-2′-deoxycytidine treatment

Cell lines were treated with 5-aza-2′-deoxycytidine (5-aza-dC, a demethylating agent) and trichostatin A (TSA, a histone deacetylase inhibitor) as previously described (7,12). Briefly, we seeded all cell lines (1×10^6^) in their respective culture medium and maintained them for 24 h before treating them with 5 *μ*M 5-aza-dC (Sigma, St. Louis, MO, USA) for 5 days and 300 nM for the final 24 h. We renewed medium containing 5-aza-dC every 24 h during the treatment and handled control cells similarly, without adding 5-aza-dC. Stock solutions of 5-aza-dC (Sigma) and TSA (Sigma) were dissolved in DMSO (Sigma) and ethanol (100%), respectively.

### Tissue samples

After obtaining institutional review board approval and appropriate informed consent, the HNSCC patients and control population (healthy subjects enrolled in a community screening study) were recruited at the Johns Hopkins School of Medicine, Department of Otolaryngology-Head and Neck Surgery. Five normal mucosa samples from healthy individuals by uvulopalatopharyngoplasty (UPPP) technique, 13 HNSCC tumors for mRNA expression array experiments, 22 salivary rinses and 14 mucosal samples from a healthy population and 33 HNSCC tumor samples were collected. Salivary rinses were obtained by brushing oral cavity and oropharyngeal surfaces with an exfoliating brush followed by rinse and gargle with 20 ml normal saline solution. The brush was gently agitated to release the obtained material into saline. Following centrifugation, the supernatant was discarded and DNA was isolated from the pellet. Tumors were snap frozen and microdissected on a cryostat to ≥75% purity. DNA from 22 salivary rinse samples and 14 normal mucosa samples from healthy individuals were analyzed as a control, to investigate the normal promoter methylation status of seven newly identified candidate genes, *MAP2K3* (mitogen-activated protein kinase kinase 3) (n=18), *MAP3K3* (mitogen-activated protein kinase kinase kinase 3) (n=18), *GNG7* (guanine nucleotide-binding protein, γ-7) (n=17), *GALNT10* (UDP-N-acetyl-α-D-galactosamine:polypeptide N-acetylgalactosaminyltransferase 10) (n=18), *PPFIBP2* (PTPRF interacting protein, binding protein 2) (n=17), *TUSC2* (tumor suppressor candidate 2) (n=16) and *TXNIP* (thioredoxin interacting protein) (n=16). The methylation status of these genes was analyzed in 33 fresh HNSCC tumor samples.

### RNA isolation, cDNA synthesis and probe hybridization

We prepared total RNA from 13 HNSCC tumors, 5 normal mucosa samples and cell lines using the RNeasy Mini kit (Qiagen, Valencia, CA, USA). Total RNA quality was checked via the Nanodrop Spectrophotometer and Agilent Bioanalyzer total RNA series II kit. Total RNA (1 *μ*g) was combined with 2 *μ*l T7 oligo(dT) primer and 2 *μ*l Poly-A controls and brought to a volume of 12 *μ*l. The samples were incubated at 70°C for 10 min. A master mix of 4 *μ*l first strand buffer, 2 *μ*l DTT and 1 *μ*l 10 mM dNTPs was added to the samples, followed by a 2-min incubation at 42°C. Superscript II (Invitrogen, Carlsbad, CA, USA) (1 *μ*l) was added to each sample and the samples were incubated at 42°C for 1 h for first strand cDNA synthesis. A master mix of 91 *μ*l water, 30 *μ*l 5X second strand buffer, 3 *μ*l 10 mM dNTPs, 4 *μ*l DNA polymerase I, 1 *μ*l *E. coli* DNA ligase and 1 *μ*l RNase H was added to each sample and the samples were incubated for 2 h at 16°C for second strand cDNA synthesis. T4 DNA polymerase (2 *μ*l) was added followed by a 5-min incubation at 16°C. EDTA (10 *μ*l, 0.5 M) was added to stop the reaction. cDNA cleanup was performed with the Affymetrix GeneChip Sample Cleanup Module, according to the manufacturer’s instructions. The cDNA was eluted in 14 *μ*l elution buffer. The final elution volume (∼12 *μ*l) was combined with 28 *μ*l of IVT mix master mix (4 *μ*l 10X IVT labeling buffer, 12 *μ*l labeling NTP mix, 4 *μ*l labeling enzyme mix, 8 *μ*l RNAse-free water) for the *in vitro* transcription cRNA synthesis reaction. The samples were incubated overnight for 16 h at 37°C followed by a hold at 4°C. Cleanup was performed as per the manufacturer’s protocol using the Affymetrix GeneChip Sample Cleanup Module. The final elution volume was ∼19 *μ*l. Concentration was checked via a nanodrop spectrophotometer. Fragmentation buffer (5X) (6 *μ*l) was then combined with 15 *μ*g cRNA and incubated at 95°C for 35 min to fragment the cRNA. The samples were ice quenched and combined with the hybridization cocktail. After 10 min of pre-hybridizing Human U133 Plus 2.0 Genome array at 45°C, 60 rpm, 200 *μ*l of cocktail was loaded onto each array and the arrays were hybridized for 16 h at 45°C, 60 rpm. The cocktail was removed and the arrays were stained and washed using the Affymetrix GeneChip Fluidics Station 450 and FS450_001 fluidics script. All arrays were scanned in the Affymetrix GeneChip Scanner 3000 and the raw analysis was performed with Affymetrix GeneChip Operating System (GCOS) 1.4. Following RNA isolation, the expertise, facilities and instrumentation for Affymetrix GeneChip experimentation were performed at the JHU microarray core facility. All reagents needed for cDNA synthesis and probe hybridization were provided by Affymetrix (Santa Clara, CA, USA)

### Microarray data analysis

mRNA gene expression profiling was performed using Affymetrix GeneChip Human Genome U133 Plus 2.0 Arrays containing 47K probe sets (Affymetrix). Signal intensity and statistical significance were established for each transcript initially using dChip version 2008 (13) and then significance analysis of microarrays (SAM) (14) software to analyze and normalize the array data. Default settings for dChip were used, including the perfect match/mismatch difference model, invariant set normalization and check single/probe/array outlier algorithm. The mRNA gene expression profiling data has been deposited in the Gene Expression Omnibus (GEO) database with the accession no. GSE29330.

### DNA extraction and bisulfite treatment

DNA was isolated as previously described (15). In brief, DNA was obtained by phenol/chloroform extraction following overnight incubation with proteinase K (Boehringer-Mannheim, Germany) at 48°C. DNA from tumor and control samples was subjected to bisulfite treatment using Epitect Bisulfite Modification kit (Qiagen) according to the manufacturer’s instructions.

### Bisulfite sequencing

Bisulfite sequence analysis was performed to determine the methylation status in the promoter regions of 126 genes obtained from gene expression profiling in normal mucosal and HNSCC tumor samples. Bisulfite-treated DNA was amplified for the 5′ region that included at least a portion of the CpG island within 1–2 kb of the first exon of 126 genes, using primer sets (data not shown). The promoter regions of the genes were found from the database of the University of California, Santa Cruz, USA (UCSC) (http://genome.ucsc.edu/). Primer sequences were determined by the MethPrimer program (16) showing the CpG islands in the promoter regions of 126 genes for bisulfite sequencing. The primers for bisulfite sequencing were designed to hybridize to regions in the promoter without CpG dinucleotides. PCR products were gel-purified using the QIAquick Gel Extraction kit (Qiagen) according to the manufacturer’s instructions. Each amplified DNA sample was sequenced by the Applied Biosystems 3700 DNA analyzer using nested, forward or reverse primers and BD terminator dye (Applied Biosystems, Foster City, CA, USA).

### Quantitative methylation-specific PCR

To determine if the methylated genes in tumor samples were cancer-specific, we investigated promoter methylation in 22 normal saliva, 14 age-matched normal mucosa from healthy individuals that were analyzed as a control, to investigate the normal promoter methylation status of seven newly identified candidate genes (*MAP3K3, MAP2K3, GNG7, GALNT10, PPFIBP2, TUSC2* and *TXNIP*) and in 33 HNSCC tumor samples by QMSP. Primer and probe sequences were determined by the MethPrimer program showing the CpG islands in the promoter regions of seven genes selected after bisulfite sequencing (Table I). Lymphocytes obtained from a healthy individual were *in vitro* methylated using excess *Sss*I methyltransferase (New England Biolabs Inc., Beverly, MA, USA) to generate completely methylated DNA that was used as a positive control standard. To quantitate the relative percent of methylation, we computed the ratio between the QMSP values of the gene of interest relative to an internal control, *ACTB*(15) (β-actin) (Table I) (gene of interest/reference gene × 100). Fluorogenic PCR was carried out in a reaction volume of 20 *μ*l consisting of 600 nM of each primer; 200 nM of probe; 0.6 U of platinum *Taq* polymerase (Invitrogen); 200 *μ*M of each dATP, dCTP, dGTP and dTTP; 1X Rox Dye reference and 1X buffer [16.6 mM of ammonium sulfate; 67 mM of Trizma (Sigma); 6.7 mM of magnesium chloride; 10 mM of mercaptoethanol; and 0.1% dimethylsulfoxide]. Thirty nanograms of bisulfite-treated DNA were used in each real-time QMSP reaction. Amplifications were carried out in 384-well plates in a 7900 Sequence Detector system (Perkin-Elmer Applied Biosystems, Norwalk, CT, USA) and were analyzed by SDS 2.3 (Sequence Detector System) (Applied Biosystems). Each reaction was performed in triplicate.

## Results

### Clinicopathological characteristics of control subjects and patients with HNSCC

Table II describes the demographic parameters of the sample populations used in this study. The mean age of normal mucosal subjects was 43.4 years (range 24–65). Forty-two percent of controls were tobacco users. Normal mucosal and tumor subjects had a similar male and Caucasian predominance. Among tumor patients, smoking rate was 78% and alcohol consumption was 69%. Tumor samples (n=33) were obtained from patients with stage I (7.4%), stage II (22%), stage III (26%) and stage IV (44%) lesions. These were from primary tumors of the oral cavity (n=9), oropharynx (n=7), hypopharynx (n=2), larynx (n=8), maxillary sinus (n=2), nasal floor (n=1), salivary gland (n=1) and unknown primary/neck (n=3). Male and Caucasian status was less prevalent in the normal salivary rinse subjects and 36% were tobacco users. Individuals from whom the normal salivary rinse was obtained were slightly younger than the population of head and neck cancer patients, with a mean age of 52.2 years (range 19–83) and 61.4 years (range 36–88), respectively. In Table III, the clinical findings of the mRNA expression cohort are given.

### Workflow of gene methylation discovery approach

**Figure 1:**
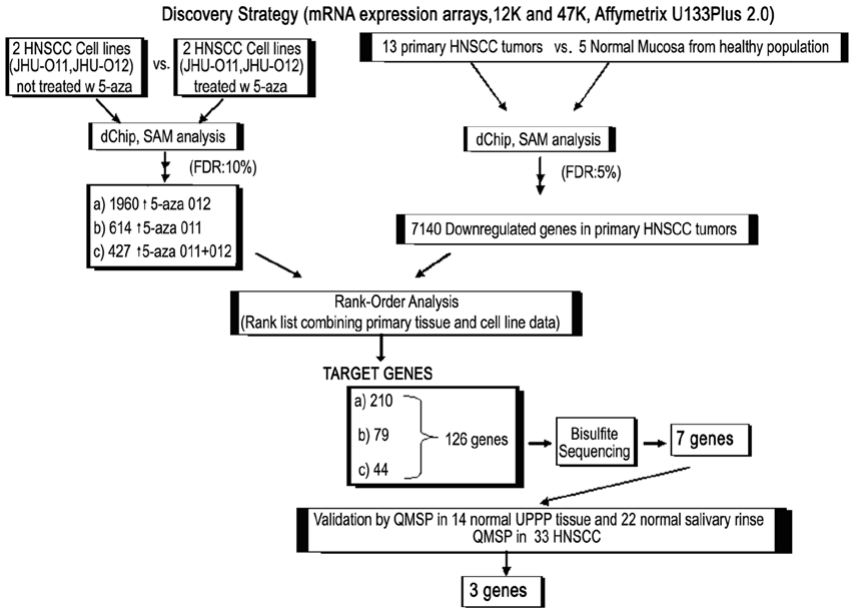
The workflow of discovery approach via gene expression array technology.

We performed pharmacological unmasking analysis on two HNSCC cancer cell lines JHU-O11 and JHU-O12 by treating cells with or without 5-aza-dC (as a control group), followed by RNA extraction and microarray analysis using Affymetrix U133 Plus 2.0. The array data were analyzed initially by dChip and then SAM. We performed a four-phase strategy to obtain the unmasked genes in the cells treated with 5-aza and downregulated genes in primary tumors. In the first phase, we compared the cell lines, either JHU-012 or JHU-011, before treatment to the cell lines treated with 5-aza, in order to identify genes that were reexpressed ≥2-fold. We found 1,960 genes that were upregulated by 5-aza-dC in the JHU-O12 cell line. SAM output was obtained at a delta value of 2.05 with a false discovery rate (FDR) of 10% and the d-score cut-off was 1.17. We found 614 reexpressed genes in 5-aza-treated JHU-O11 (SAM output; delta=2.089, FDR=10%, d-score cut-off=2.8); 427 genes were commonly upregulated in both cell lines when the cell lines were normalized and analyzed together (SAM output; delta=1.44, FDR=10%, d-score cut-off=1.88) (Fig. 1). In the second phase of our analysis, we further extracted RNA and performed the 47K mRNA expression array analysis on 13 primary HNSCC tumors and 5 normal mucosal samples from non-cancer control patients. Following initial dChip and SAM analysis (SAM output; delta=1.247, FDR=10%, d-score cut-off=0.24), we found 7,140 downregulated genes in primary HNSCC tumors compared with normal mucosa. In the third phase, we investigated the three data sets (Fig. 1) below: a) SAM output of 1,960 upregulated genes after 5-aza treatment of JHU-012 vs. SAM output of 7,140 downregulated genes in primary HNSCC. We found that 210 genes that were upregulated by 5-aza-dC in the JHU-O12 cell line and showed downregulation in tumor samples. b) SAM output of 614 upregulated genes after 5-aza treatment of JHU-011 vs. SAM output of 7,140 downregulated genes in primary HNSCC. We found 79 genes that were upregulated by 5-aza-dC in the JHU-O11 cell line and showed downregulation in tumor samples. c) SAM output from analyzing both cell lines together in the same SAM computation, of 427 upregulated genes after 5-aza treatment of JHU-011 and JHU-012 vs. SAM output of 7,140 downregulated genes in primary HNSCC. We found 44 genes that were upregulated by 5-aza-dC in the JHU-O11 and JHU-012 cell lines and showed downregulation in tumor samples, suggesting that methylation might be involved in gene downregulation.

**Figure 2:**
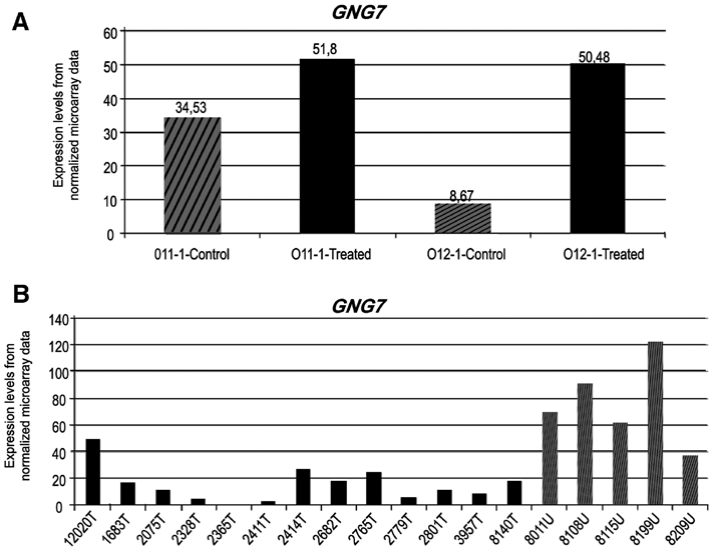
Expression array values from normalized microarray results of the *GNG7* gene: (A) from cell lines treated with 5-aza and non-treated with 5-aza; (B) human tumors and normal tissue samples.

**Figure 3:**
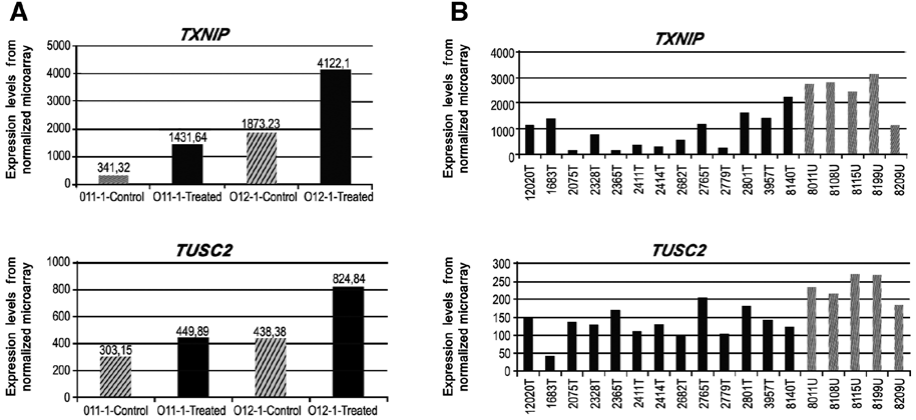
Expression array values from normalized microarray results of the *TXNIP* and *TUSC2* genes: (A) from cell lines treated with 5-aza and non-treated with 5-aza; (B) human tumors and normal tissue samples.

In the fourth phase of our strategy, we rank-ordered the results of upregulated genes obtained from these 3 data sets and found 126 common genes. We then examined promoter regions of the 126 genes for CpG islands and performed bisulfite sequencing analysis of the promoter region of these genes. We found that seven genes showed a differential methylation pattern between normal and neoplastic samples (Fig. 1). Table IV shows the d-scores, fold change and q-values of these genes by SAM analysis in human and cell line samples. After validation of these genes in a cohort of 33 HNSCC patients and normal salivary and mucosal samples from healthy individuals by QMSP, we found 3 genes of interest (*GNG7*, *TXNIP* and *TUSC2*). The upfold expression arrays of these genes are given in Figs. 2 and 3. A 1.5-fold upregulation in *GNG7* expression was observed in 5-aza-treated JHU-011, whereas the rate of change was 5.8-fold in 5-aza-treated metastatic JHU-012 cells (Fig. 2). The *TXNIP* gene was upregulated 4.19-fold in JHU-011 cells, whereas a 2.2-fold change was observed in the JHU-012 cells. The *TUSC2* gene was upregulated in a similar manner in both cell lines (Fig. 3).

### Genes specifically methylated in HNSCC tumors

**Figure 4:**
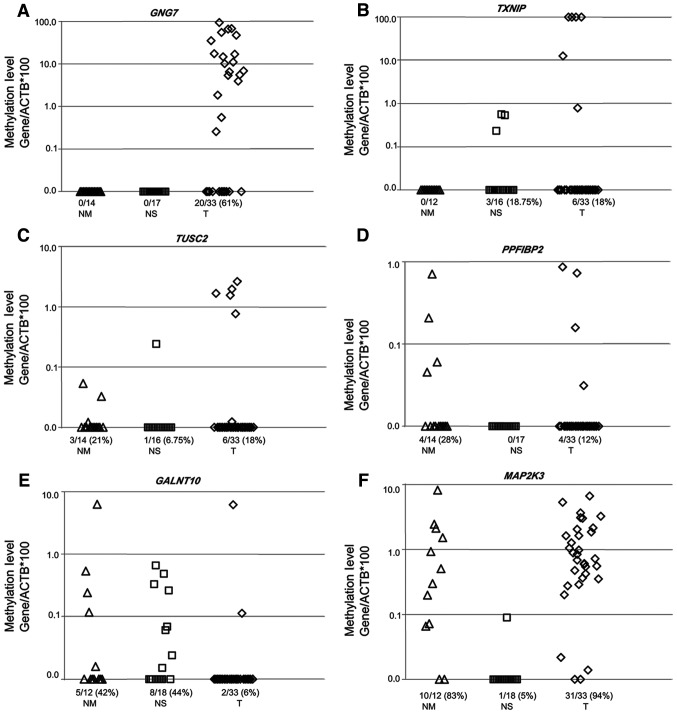
Scatter graph displaying the distribution of the methylation levels of 6 candidate genes: (A) *GNG7*; (B) *TXNIP*; (C) *TUSC2*; (D) *PPFIBP2*; (E) *GALNT10*; (F) *MAP2K3* in normal mucosa samples (NM), normal salivary rinses (NS) and HNSCC tumors (T).

We then performed bisulfite sequencing analysis on the promoter region of 126 genes as described above in our discovery approach using 4 normal mucosal and 4 HNSCC samples. Seven genes (*GNG7, GALNT10, TXNIP, TUSC2, PPFIBP2, MAP2K3* and *MAP3K3*) were found to have no methylation in normal mucosal samples but displayed high methylation frequency in HNSCC samples (Table V). This high specificity prompted us to further investigate the methylation frequency in a larger cohort of normal mucosal and HNSCC specimens. We then performed QMSP on 22 saliva and 14 normal mucosal samples from healthy individuals and 33 HNSCC tumor samples for seven genes selected. The *GNG7* gene showed no methylation in normal mucosal samples (0/14) and normal salivary rinses (0/17). The methylation rate was 61% (20/33) on the promoter region of the *GNG7* gene in primary HNSCC tumor samples and these tumors harbored high methylation values, mostly between 10 and 100%. *TXNIP, TUSC2, PPFIBP2, GALNT10* and *MAP2K3* demonstrated varying degrees of methylation on their promoter regions in normal mucosa, normal salivary rinses and HNSCC tumor samples respectively (Fig. 4). We observed no methylation in the promoter region of *MAP3K3* [0/14 (0), 0/18 (0) and 0/33 (0)] gene in normal mucosa, normal salivary rinses and HNSCC tumor samples, respectively.

## Discussion

In the present study, we combined a proven pharmacologically demethylating-unmasking strategy with an expanded 47K expression microarray platform to identify novel cancer-specific methylated genes. Among the 47,000 transcripts of the Affymetrix Human Genome U133 Plus 2.0 expression arrays, we first identified seven genes of interest. We then performed QMSP on 22 saliva and 14 mucosal samples from healthy individuals and 33 HNSCC tumor samples for the seven genes selected and verified 3 genes showing hypermethylation in the promoter regions of HNSCC tissues and minimal or absent methylation in normal salivary rinses and mucosal samples. The *GNG7* gene was the most marked, showing no methylation in normal mucosal samples and normal salivary rinses respectively, while 61% of primary HNSCC tumor samples were methylated. *TXNIP* methylation values were less than 1% in normal salivary rinses and the promoter was not methylated in normal mucosal samples, whereas the methylation levels were over 10% in tumors. Similarly, the *TUSC2* gene was found to be methylated in 0.1% of normal mucosa samples and showed almost no methylation (under 1%) in normal salivary rinses. Other genes were not found to be useful as potential biomarkers.

We found that the guanine nucleotide-binding protein, γ-7 (*GNG7*) promoter was specifically methylated in HNSCC. *GNG7* is located on chromosome 19 and is a member of the guanine nucleotide-binding proteins (G proteins) which are involved as a modulator or transducer in various transmembrane signaling systems. The β and γ chains are required for the GTPase activity, for replacement of GDP by GTP and for G protein-effector interaction; they are important in the regulation of adenylyl cyclase signaling in certain regions of the brain and have a role in the formation or stabilization of a G protein heterotrimer [G(olf) subunit α-β-γ-7] that is required for adenylyl cyclase activity in the striatum. *GNG7* showed homozygous deletions in cell lines of classical Hodgkins lymphoma (17). Decreased expression of *GNG7* identified by Ray *et al*(18) was confirmed in pancreatic malignancies (19) and esophageal cancer (20). Expression of G-γ-7 mRNA was downregulated in extrahepatic cholangiocarcinoma (EHCC) tissue compared to pericancerous bile duct and normal bile duct tissues and in poorly differentiated EHCC tissues (21).

Thioredoxin-interacting protein is encoded by the *TXNIP* gene and interacts with thioredoxin and ZBTB32. This gene functions as an oxidative stress mediator by inhibiting thioredoxin activity or by limiting its bioavailability and acts as a transcriptional repressor, between transcription factors and co-repressor complexes and its overexpression induces G0/G1 cell cycle arrest and is necessary for the maturation of natural killer cells (22–25).

Tumor suppressor candidate 2 is encoded by the *TUSC2* gene which is a highly conserved lung cancer candidate gene (26,27). In malignant pleural mesothelioma (28) and nasopharyngeal carcinoma (29), expression of the *TUSC2* gene was found to be downregulated. *TUSC2* is often deleted in lung, breast, head and neck, renal and other types of cancer (30) and was reported to be methylated in human lung cancer cells (31). Furthermore, large-scale analysis of *TUSC2* expression in lung cancer and in bronchial squamous metaplastic and dysplastic lesions showed reduced expression levels of *TUSC2* compared to normal hyperplastic epithelia, indicating it could be an early event in cancer progression (32).

It is known that the cell culture may influence DNA methylation and present larger stretches of methylation events and some drawbacks when compared to primary tumors (33). Therefore, as a future step, this issue may be investigated by use of a larger panel of HNSCC cell lines. However, this fact does not invalidate our findings and the data of our study showing that the *GNG7* gene is a promising candidate tumor suppressor gene and biomarker for HNSCC.

It would be helpful to test independent cohorts to evaluate the utility of these genes as HNSCC biomarkers. Additional studies in larger, prospective cohorts would also determine the prognostic significance of detection of these markers in saliva, serum or plasma samples from HNSCC patients.

## Figures and Tables

**Table I:** Primers and probe sequences of selected genes for validation by QMSP.

Gene	Probe 5′-3′ (6-FAM-5′-3′-6-TAMRA)	Forward 5′-3′ (primer)	Reverse 5′-3′ (primer)
*ACTB*	ACCACCACCCAACACACAATAACAAACACA	TGGTGATGGAGGAGGTTTAGTAAGT	AACCAATAAAACCTACTCCTCCCTTAA
*MAP3K3*	GGGAGTCGGGCGTTGTTTCGATG	ATGCGTAGAGGCGGGGGTTT	ACACGATAAACCAATCCCGCC
*MAP2K3*	GGGCGACGTTTGTTGGCGTTAGG	CGTGTTGTTTCGTTATCGGGTA	AACTATCTCCCGACGCTACTC
*GNG7*	GCGCGGGATTCGAATTCGCGAAAT	CGGAGTTGGTATGTAGGATTCG	CCCCGACTACGAAAAACCGAA
*GALNT10*	CGTTTCGGTTCGGTATTTTGTAGCG	TCGTAAAGTTTTAGAGGGCGG	AATCTCTACGCTACAAACTCGA
*PPFIBP2*	ACGAGGTAGGTTCGAAGGGGCG	TAATCGGAGTTGTGCGGAGGA	CCTATTCCCGAAAAACCGACC
*TUSC2*	CGGAAGCGGAAGTGAGGTTTTCGT	AGGGCGTTTATTGGTTTCGTTT	CGCAATCCGCACTACCATAAC
*TXNIP*	CGAGGGTAGTACGAGTTTTCGGGT	GCGATTTTATTGATTGGTCGGG	CGTCTCTATATAATAACCCGAACC

**Table II:** QMSP results and demographics of the patients with HNSCC.

Tumor sample	*GNG7*	*TXNIP*	*TUSC2*	*PPFIBP2*	*GALNT10*	*MAP2K3*	Age	Gender^a^	Race^a^	Smoking	Alcohol	Tumor site	Stage
1	**N**	**N**	**N**	**N**	**N**	**Y**	67	M	C	Yes	Yes	Nasal floor	2
2	**N**	**N**	**Y**	**N**	**N**	**Y**	57	M	C	Yes	Yes	Larynx	2
3	**N**	**N**	**N**	**Y**	**N**	**Y**	61	M	C	No	No	Neck	3
4	**Y**	**Y**	**N**	**N**	**N**	**Y**	60	F	C	Yes	Yes	Larynx	4
5	**N**	**N**	**N**	**N**	**N**	**Y**	55	M	AA	Yes	Yes	Larynx	2
6	**Y**	**N**	**N**	**N**	**N**	**Y**	54	M	C	No	Yes	Oropharynx	4
7	**Y**	**Y**	**N**	**N**	**N**	**Y**	64	M	AA	Yes	Yes	Hypopharynx	3
8	**Y**	**N**	**N**	**N**	**N**	**Y**	55	F	A	Yes	No	Oral cavity	1
9	**Y**	**Y**	**N**	**N**	**N**	**N**	80	M	C	Yes	No	Oral cavity	
10	**Y**	**N**	**N**	**N**	**N**	**Y**	54	F	C	No		Oral cavity	4
11	**N**	**Y**	**Y**	**Y**	**N**	**Y**	62	M	C	Yes	Yes	Oropharynx	4
12	**N**	**N**	**N**	**N**	**N**	**N**	72	M	C	Yes	Yes	Hypopharynx	3
13	**N**	**N**	**N**	**N**	**N**	**Y**	42	M	C	Yes	No	Larynx	2
14	**Y**	**Y**	**Y**	**N**	**N**	**Y**	66	M	C	Yes	No	Oropharynx	4
15	**N**	**N**	**N**	**N**	**N**	**Y**	74	M	C	Yes		Larynx	2
16	**Y**	**N**	**N**	**N**	**N**	**Y**	58	M	A	Yes	Yes	Oropharynx	4
17	**Y**	**N**	**N**	**N**	**Y**	**Y**	56	F	C	Yes	Yes	Oral Cavity	2
18	**Y**	**N**	**N**	**N**	**N**	**Y**	43	M	C	Yes	Yes	Oropharynx	4
19	**Y**	**N**	**N**	**N**	**N**	**Y**	68	M	C	Yes	Yes	Oropharynx	4
20	**Y**	**N**	**N**	**N**	**N**	**Y**	63	M	A	Yes		Oral cavity	
21	**Y**	**N**	**N**	**N**	**N**	**Y**	64	F	C	No	Yes	Oral cavity	3
22	**N**	**N**	**N**	**N**	**N**	**Y**	88	M	C	Yes	Yes	Oral cavity	
23	**Y**	**N**	**N**	**N**	**N**	**Y**	42	M	C	Yes	No	Oral cavity	3
24	**N**	**N**	**N**	**N**	**N**	**Y**	51	M	C	Yes	Yes	Larynx	4
25	**N**	**N**	**Y**	**N**	**N**	**Y**	80	M	C	No	Yes	Neck	
26	**Y**	**N**	**Y**	**N**	**N**	**Y**	58	M	C	Yes	Yes	Larynx	3
27	**Y**	**N**	**N**	**N**	**N**	**Y**	71	M	C	Yes	Yes	Neck	
28	**N**	**N**	**Y**	**N**	**N**	**Y**	48	M	C	Yes	Yes	Oropharynx	4
29	**Y**	**N**	**N**	**N**	**N**	**Y**	61	M	C	Yes	No	MS^b^	1
30	**Y**	**N**	**N**	**Y**	**N**	**Y**	77	M	C	No	No	Salivary gland	
31	**Y**	**N**	**N**	**Y**	**N**	**Y**	67	M	C			Larynx	3
32	**Y**	**N**	**N**	**N**	**N**	**Y**	36	M	C	Yes	No	Oral Cavity	4
33	**N**	**Y**	**N**	**N**	**Y**	**Y**	74	F	C	No	Yes	MS^b^	4

Y, methylated; N, unmethylated.

a M, male; F, female; C, Caucasian; A, African; AA, African-American.

b MS, maxillary sinus.

**Table III:** Clinical characteristics of mRNA expression array cohort.

Case	Diagnosis	Age	Gender^a^	Race^a^	Overall stage	T	N	M	Site	Tobacco	Alcohol
1	Normal	20	M	C	NA	NA	NA	NA	Left tonsil	No	No
2	Normal	28	M	C	NA	NA	NA	NA	Right tonsil	No	No
3	Normal	28	M	C	NA	NA	NA	NA	Uvula	No	No
4	Normal	28	M	C	NA	NA	NA	NA	Left tonsil	NA	NA
5	Normal	30	M	C	NA	NA	NA	NA	Uvula	No	No
6	Cancer	62	M	C	3	3	0	0	Larynx	No	Yes
7	Cancer	80	F	C	1	1	2A	0	Oral cavity	No	NA
8	Cancer	NA	NA	NA	NA	NA	NA	NA	NA	NA	NA
9	Cancer	82	M	C	3	3	0	0	Larynx	Yes	NA
10	Cancer	74	M	C	NA	NA	NA	NA	NA	NA	NA
11	Cancer	71	M	C	4	4	0	0	Oral cavity	Yes	No
12	Cancer	56	M	AA	NA	NA	NA	NA	NA	NA	NA
13	Cancer	62	F	AA	1	1	0	0	Oral cavity	Yes	Yes
14	Cancer	58	M	C	3	2	2B	0	Oropharynx	No	Yes
15	Cancer	61	M	C	NA	NA	NA	NA	NA	NA	NA
16	Cancer	89	M	C	NA	NA	NA	NA	NA	Yes	Yes
17	Cancer	63	M	C	2	2	0	0	Larynx	Yes	Yes
18	Cancer	50	F	C	4	3	2B	0	Oropharynx	Yes	Yes

a M, male; F, female; C, Caucasian; A, African; AA, African-American; NA, not available. T, tumor; N, node; M, metastasis.

**Table IV:** The results of SAM analysis in human and cell line specimens.

Downregulated genes from the SAM output of human tumor vs. normal mucosal tissues					Upregulated genes from the SAM analysis of 5-aza non-treated vs. 5-aza-treated JHU-011 + JHU-012 cells				
Rank	**Gene ID**	**Score (d)**	**Fold change**	**q-value (%)**	Rank	**Gene ID**	**Score (d)**	**Fold change**	**q-value (%)**
5506	*GALNT10*	**−2.84**	**0.55**	3.37	61	*GALNT10*	**8.38**	**2.28**	2.55
5189	*MAP2K3*	**−2.92**	**0.65**	2.89	13	*MAP2K3*	**12.07**	**2.55**	0.00
3406	*MAP3K3*	**−3.44**	**0.73**	1.47	309	*MAP3K3*	**5.15**	**2.12**	7.68
1939	*PPFIBP2*	**−3.99**	**0.58**	0.86	389	*PPFIBP2*	**4.75**	**1.99**	9.28
					Upregulated genes from the SAM analysis of non-treated vs. 5-aza-treated JHU-012 cell lines				

34	***GNG7***	**−8.32**	**0.21**	0.00	313	***GNG7***	**13.95**	**5.34**	4.02
813	***TUSC2***	**−4.82**	**0.57**	0.37	408	***TUSC2***	**12.02**	**1.44**	4.56
					Upregulated gene from the SAM analysis of non-treated vs. 5-aza-treated JHU-011 cell lines				

1317	***TXNIP***	**−4.32**	**0.36**	0.61	362	***TXNIP***	**9.55**	**5.39**	7.40

**Table V:** Methylation analysis of candidate genes in 4 normal mucosa and 4 tumor samples by bisulfite sequencing.

Probe name	Gene ref ID	Gene name	Chromosome location	Normal mucosa tissue, n (%)	HNSCC tumor tissue, n (%)
215499_at	NM_145109	*MAP2K3*	chr17:21,128,561–21,159,144	0 of 4 (0)	4 of 4 (100)
203514_at	NM_002401	*MAP3K3*	chr17:59,053,533–59,127,402	0 of 4 (0)	4 of 4 (100)
220296_at	NM_198321	*GALNT10*	chr5:153,550,488–153,780,003	0 of 4 (0)	4 of 4 (100)
201010_s_at	NM_006472	*TXNIP*	chr1:144,149,819–144,153,985	0 of 4 (0)	4 of 4 (100)
203273_s_at	NM_007275	*TUSC2*	chr3:50,337,345–50,340,672	0 of 4 (0)	4 of 4 (100)
212841_s_at	NM_003621	*PPFIBP2*	chr11:7,491,577–7,631,567	0 of 4 (0)	2 of 4 (50)
206896_s_at	NM_052847	*GNG7*	chr19:2,462,218–2,653,746	0 of 4 (0)	1 of 3 (33)
